# It's Harder to Push, When I Have to Push Hard—Physical Exertion and Fatigue Changes Reasoning and Decision-Making on Hypothetical Moral Dilemmas in Males

**DOI:** 10.3389/fnbeh.2018.00268

**Published:** 2018-11-23

**Authors:** Matthias Weippert, Michel Rickler, Steffen Kluck, Kristin Behrens, Manuela Bastian, Anett Mau-Moeller, Sven Bruhn, Alexander Lischke

**Affiliations:** ^1^Institute of Sport Science, Faculty of Philosophy, University of Rostock, Rostock, Germany; ^2^Institute of Philosophy, Faculty of Philosophy, University of Rostock, Rostock, Germany; ^3^Department of Physiotherapy, ISBA University of Cooperative Education, Schwerin, Germany; ^4^Department of Clinical Chemistry and Laboratory Medicine, University Medicine Rostock, Rostock, Germany; ^5^Department of Psychology, University of Greifswald, Greifswald, Germany

**Keywords:** moral judgment, exhaustion, stress, cortisol, utilitarian moral, strenuous exercise, cognition, effort

## Abstract

Despite the prevalence of physical exertion and fatigue during military, firefighting and disaster medicine operations, sports or even daily life, their acute effects on moral reasoning and moral decision-making have never been systematically investigated. To test the effects of physical exertion on moral reasoning and moral decision-making, we administered a moral dilemma task to 32 male participants during a moderate or high intensity cycling intervention. Participants in the high intensity cycling group tended to show more non-utilitarian reasoning and more non-utilitarian decision-making on impersonal but not on personal dilemmas than participants in the moderate intensity cycling group. Exercise-induced exertion and fatigue, thus, shifted moral reasoning and moral decision-making in a non-utilitarian rather than utilitarian direction, presumably due to an exercise-induced limitation of prefrontally mediated executive resources that are more relevant for utilitarian than non-utilitarian reasoning and decision-making.

## Introduction

“Of course, I would have had to foul him.” Philipp Lahm, the German national player, took this memorable slogan immediately after the semi-final match of an important international Soccer Championship. Obviously, he was sure that a foul would have been the right choice in a match situation when an opponent had run away from him and could prepare the equalizer goal for the opposing team^1^. After the match, he apologized for his decision not to foul because fouling his opponent would have prevented a goal against his team. Apparently, Lahm would have made a different decision after the match, when he was no longer under physical exertion and had sufficient time to weigh up the costs and benefits of fouling. Although this situation may not be a perfect example of a moral dilemma, it nevertheless shows that physical exertion and fatigue may affect moral reasoning and moral decision-making. However, the effects of physical exertion and fatigue on moral reasoning and moral decision-making have never been investigated before. This is surprising because many individuals have to solve moral dilemmas under conditions of physical exertion and fatigue, for example during military, firefighting or disaster medicine operations, and sometimes also in sport. Assessing the effects of physical exertion and physical fatigue on moral reasoning and moral decision-making is, thus, not an academic exercise but of practical importance. As fatigue limits not only physical but also cognitive functions (Van der Linden et al., 2003; Dietrich, 2006; Ishii et al., 2014; Enoka and Duchateau, 2016; Schmit and Brisswalter, 2018), it seems plausible to assume that fatiguing exercise may also affect performance on a moral dilemma task. Dual process theories suggest that cognitively demanding decisional tasks can be solved on basis of two processes: slow processes that involve deliberative thinking and fast processes that involve intuitive thinking (Baumeister et al., 1998; Kahneman, 2003; Evans, 2008; Rand, 2016). Fatigue-induced limitations of executive resources and executive functioning have been shown to impair deliberative and to facilitate intuitive thinking, indicating that similar effects can be expected during moral reasoning and moral decision-making. Accordingly, it has been shown that cognitive fatigue affects performance on a moral dilemma task in the proposed manner (Timmons and Byrne, 2018). Whereas non-fatigued participants favored a dilemma solution that involved the killing of one individual in favor of saving five other individuals (utilitarian reasoning), fatigued participants favored a dilemma solution that did not involve the killing of one individual in favor of saving five other individuals (non-utilitarian or deontological reasoning). These findings are also in accordance with findings on decision-making in the context of cooperation, distributive justice, and social choice theories, which show that cognitive fatigue facilitates intuitive and limits deliberative decision-making (Baumeister et al., 1998; Capraro and Cococcioni, 2016; Rand, 2016). Utilitarian reasoning and utilitarian decision-making involves the transgression of one of the most fundamental moral principles, namely, that it is wrong to harm or even kill another individual (Cushman et al., 2012; Miller et al., 2014; Reynolds and Conway, 2018). As a consequence, utilitarian decision-making, which violates this principle, involves deliberative thinking, while non-utilitarian decision-making, which follows this principle, involves intuitive thinking. It remains to be investigated, whether physical fatigue affect moral reasoning and moral decision making in a similar manner as cognitive fatigue. In this regard, it is important to note that physical fatigue does not automatically follow physical exertion. Depending on its type, duration and intensity, physical exercise and exertion may either alleviate fatigue, thereby facilitating prefrontally mediated executive resources and executive functioning, or enhancing fatigue, thereby impairing prefrontally mediated executive resources and executive functioning (Brisswalter et al., 2002; Dietrich, 2006; Young and Koenigs, 2007; Audiffren et al., 2009; Forbes and Grafman, 2010; Dietrich and Audiffren, 2011; Labelle et al., 2013; McMorris, 2016; Schmit and Brisswalter, 2018).

It may, thus, be possible that, moderated via physical exertion and fatigue, moral reasoning and moral decision-making vary as a function of the type, intensity, and duration of the physical exercise. To test this possibility, we investigated how moderate and high intensity exercise affected participants' performance on a task that involved reasoning and decision-making on hypothetical moral dilemmas. Although hypothetical reasoning and decision-making does not necessarily have to mirror real reasoning and decision-making (Amir et al., 2012; FeldmanHall et al., 2012), the dilemma task was the only possible task that allowed us to test our hypotheses about life and death decisions in an ethical way. During the task, participants had to reason in form of ratings and to make decisions in form of choices in the context of established dilemmas that had been modified as suggested by recent criticism (Christensen and Gomila, 2012; Christensen et al., 2014). Participants performed the task either during moderate or high exertion, which was induced by >20 min of cycling on an ergometer. Of note, moderate and high exertion was induced on basis of individually-tailored ergometer workloads because individual responses to the same absolute workload can differ dramatically in terms of exertion and fatigue (Demello et al., 1987; Marcora, 2010; Mann et al., 2013). To assess participants' physical exertion and fatigue, we employed a battery of psychological (e.g., perceived effort, fatigue, mood) and physiological (e.g., heart rate and blood lactate concentration) measures. On basis of previous findings (Timmons and Byrne, 2018), we expected participants to show less utilitarian reasoning and less utilitarian decision-making after high as compared to moderate intensity exercise, presumably due to acute exertion- and fatigue-induced changes from deliberative to intuitive thinking during task performance.

## Methods

### Participants

In order to determine the number of participants that we needed to detect meaningful differences in moral reasoning and moral decision-making in our analyses, we performed a power analysis with G^*^Power (Faul et al., 2007). However, exertion or fatigue-induced differences in moral reasoning and moral decision-making have not been investigated before, indicating the novelty of our study. We, thus, based our power analysis on differences in executive functioning that have been reported after 20 min of maximal intensity exercise in previous studies (Chang et al., 2012). G^*^Power indicated that we had to recruit a minimum of 24 participants for our study (1-β: 0.80, α: 0.20, *f* : 0.25). Considering a potential dropout of 30%, we recruited 32 participants from local soccer clubs by public announcements. To limit sample size without reducing the statistical power due to possible gender effects on moral judgment and decision-making (Fumagalli et al., 2010; Friesdorf et al., 2015; Capraro and Sippel, 2017) we defined, *a priori*, a male population to be represented by our sample. Male participants that were aged between 18 and 35 years and that were well-educated (Abitur) were included in the study. Participants that were in psychotherapeutic or psychopharmacological treatment at the time of the study and participants that suffered from cardiovascular or orthopedic disorders that speak against participation in an exhausting exercise at the time of the study were excluded. Inclusion and exclusion of participants was determined on basis of an established screening procedure (Lischke et al., 2018). All participants were asked to refrain from any exhaustive exercise 48 h and from caffeine and alcohol 12 h prior to the experimental sessions. At the beginning of the experimental session, participants were randomly assigned to a moderate intensity (*n* = 16) or a high intensity (*n* = 16) ergometer cycling intervention. Of note, participants were only informed that the aim of the experiment was to investigate reasoning and decision-making in social contexts. They were, thus, left naïve to the particular purpose of the moral dilemma task. After completion of the experiments, participants were fully debriefed about the aims of the study. The study was approved by the local ethics committee of the University of Rostock (approval number: A 2017-0034). Written-informed consent was obtained from all participants.

### Procedure

**Figure 1:**
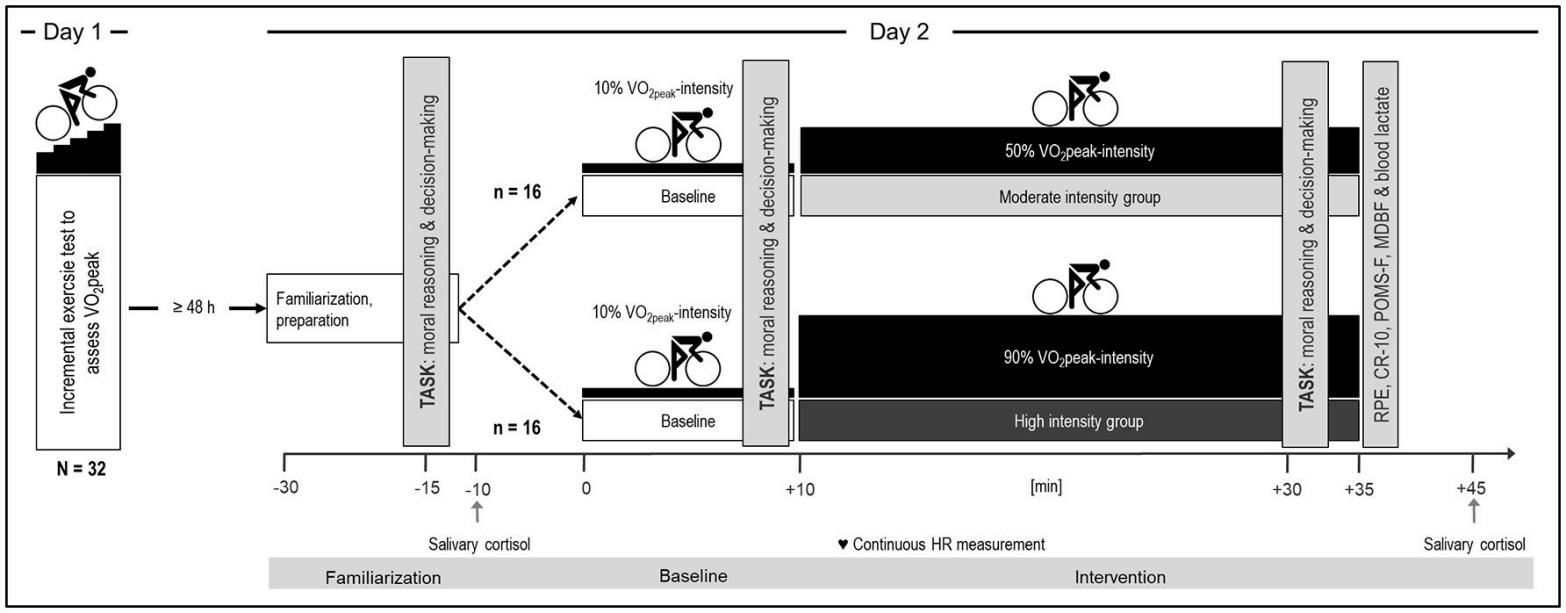
Experimental setup of the study.

Participants were invited to two experimental sessions that took place on two separate days: a preparation day (day one) and a testing day (day two, see Figure 1). To avoid carry-over effects of exhausting exercise, a minimum of 48 h were scheduled between the preliminary assessment on day one and the experimental session on day two.

On day one, participants' cardiorespiratory fitness was assessed. Therefore, participants performed an incremental cycling test on an electromagnetically braked bicycle ergometer (SRP 3000, Sportplus Germany) until exhaustion, starting at a power output of 50 W with a stepwise increase of 25 W·min^−1^. The incremental test was stopped if cycling cadence dropped below 60 rpm and participants were no longer able to maintain the required power output despite strong verbal encouragement. The following physiological and psychological indicators of participants' end-exercise exertion were collected: heart rate (HR; Polar^®^ RS800 heart rate monitor, Polar Inc., Finland) and volumes of the respiratory gases oxygen (VO_2_) and carbon dioxide (VCO_2_) were permanently measured using a metabolic measuring system (Metamax 3B, Cortex Medical, Germany) to quantify cardiovascular strain and metabolic demands. Further, measured peak oxygen uptake (VO_2peak_) was normalized to participants' bodyweight (relative VO_2peak_) to analyse the cardio-respiratory fitness of each participant. Blood lactate concentration (LactateScout^®^, Senslab, Germany), also reflecting metabolic demands and exercise intensity, was determined 1 min after exercise cessation. Individual peak HR (HR_peak_) and power output (P_peak_) at VO_2peak_ were used to determine and evaluate individual exercise intensity levels for the experimental session on day two.

On day two, participants were prepared and familiarized with the experimental setup and the moral dilemma task. After the familiarization procedure, participants cycled on the ergometer (SRP 3000, Sportplus Germany) in front of a custom-made computer system that was used for the administration of the moral dilemma task. During the baseline and intervention periods participants were instructed to continuously cycle at two different intensity levels. Ten minutes of cycling at a low power output, corresponding to 10% of participants' VO_2peak_, served as individual baseline condition. Low intensity cycling was used as individual baseline because dual tasking (here: cycle intervention and moral dilemma task) *per se* may have an impact on participants' performance on the moral dilemma task. Baseline cycling was then followed by the intervention: Depending on participants' assignment to the intervention groups, they either cycled at a moderate (50% of VO_2peak_) or a high (90% of VO_2peak_) power output for 25 min. Between the 20th and 25th min of cycling at moderate or high intensity, participants performed the moral dilemma task. Exercise duration was limited to a duration of 25 min because cycling at an intensity of ≥90% of VO_2peak_ requires strong effort. It progressively induces high levels of exertion and fatigue, which normally leads to exercise termination within 30 min in healthy young males (Billat et al., 2000).

### Physiological measures

To determine participants' physiological state in terms of cardiorespiratory strain and autonomic activation during baseline, moderate, and high intensity cycling HR was continuously recorded. Average absolute HR as well as normalized HR (% HR: percentage of the individual peak HR achieved during the initial all-out incremental exercise test) were calculated for the different experimental periods. Further, capillary blood samples were collected from the earlobe within the first min after the cessation of moderate and high intensity cycling to determine participants' blood lactate concentration. Salivary cortisol was collected 10 min before (baseline) and 10 min after (intervention) cycling (Dickerson and Kemeny, 2004). Salivary samples were analyzed using Cobas^®^ e411 (Roche Diagnostics, Switzerland) and CORT II reagent (Ref.06687733, Roche Diagnostics, Switzerland) according to the manufactures protocol.

### Psychological measures

To measure participants' psychological state after the intervention, the following self-report scales were immediately administered after exercise cessation. Perceived effort was rated on a Borg-Scale (Borg, 1990; Marcora, 2010; Pageaux, 2016) that ranged from 6 (no effort at all) to 20 (maximal effort). Perceived pain of the working muscles (CR-10, Cook et al., 1997) was assessed on a numeric pain scale that ranged from 0 (no pain at all) to 10 (extremely intense pain, almost unbearable). Perceived state fatigue was evaluated with the Profile of Mood State subscale fatigue (POMS-F, Albani et al., 2005) that ranged from 0 (no fatigue at all) to (maximal fatigue). Mood, wakefulness and arousal were assessed with the Multidimensional Mood State Questionnaire (MDMQ, Hinz et al., 2012). The mood scale had a range from 4 (positive) to 20 (negative), the wakefulness had a range from 4 (tired) to 20 (awake), and the arousal scale had a range from 4 (calm) to 20 (aroused). Moral orientation (idealism, relativism) was assessed with the ethical position questionnaire (EPQ, Forsyth, 1980) that had a range from 0 (never) to 4 (always). Psychopathological distress was assessed with the Brief Symptom Inventory (BSI, Franke et al., 2017) that had a range from 0 (not at all) to 4 (very strong).

### Moral dilemma task

DMDX was used for dilemma presentation and response registration (Forster and Forster, 2003). The task comprised 8 moral dilemmas (Table 1) that were modified versions of dilemmas that had been used in previous studies (Greene et al., 2001, 2008; Koenigs et al., 2007; Moore et al., 2008). Two types of a moral dilemma were used, which differed in their directedness of harm. Whereas in a *personal* dilemma the agent is directly involved in the production of the proposed harm (e.g., by pushing someone hard with ones' arms), in an *impersonal* dilemma the agent is only indirectly involved in the production of the proposed harm (e.g., by pushing a button with ones' finger). Vivid language was used to describe each dilemma from a first person perspective, that is, participants' perspective, in 171 words across four paragraphs. The first paragraph described the setting and emphasized that the life of several individuals was in danger. The second paragraph emphasized that the life of five individuals could be saved by sacrificing the life of one individual through a distinct action. The third paragraph asked participants to rate the moral permissibility of the described action (moral reasoning) on a rating scale that ranged from 1 (morally permissible) to 9 (morally not permissible). The fourth paragraph asked participants to either engage or refrain from the action (moral decision-making). Participants' ratings, decisions, and decision times were recorded throughout the presentation of the dilemmas, which was pseudo-randomized and counter-balanced across participants and conditions. Following recent suggestions, the dilemmas were closely matched on criteria that have been shown to affect moral reasoning and moral decision-making (Christensen and Gomila, 2012; Christensen et al., 2014), such as differences in personal force of the protagonist (personal vs. impersonal), differences in evitability of death of the to be sacrificed individual (avoidable vs. unavoidable), differences in the trade-off between the number of saved and sacrificed individuals (one vs. five), or differences in the framing of the questions (saving vs. killing). Besides this, the dilemmas were put into a soccer context to make them more realistic and more appealing for our participants, who were soccer fans. Semantic examples of the applied dilemmas are given in the Supplementary Material. It should be noted, however, that the dilemmas were translated from German into English. The English dilemmas may not fully capture the aspects of the German dilemmas, indicating that they should not be used in further studies, particularly because language-related properties can affect performance on moral dilemma tasks (Christensen et al., 2014; Costa et al., 2014).

**Table 1:** Moral dilemmas.

**Set**	**Modified scenario**	**Original scenario**	**Type of scenario**
A	(1) Exit	Burning building	Personal
	(2) Box	Footbridge	Personal
	(3) Lift	Mineshaft	Impersonal
	(4) Staircase	Submarine	Impersonal
B	(1) Gate	Submarine	Personal
	(2) Firecrackers	Shooting	Personal
	(3) Explosion	Cinderblock	Impersonal
	(4) Trolley	Trolley	Impersonal

In line with recent recommendations regarding the analysis of fatigue related effects (Enoka and Duchateau, 2016), we performed a change score analysis of the moral dilemma data. Individual change from baseline was calculated for moral reasoning and moral decision-making (baseline score – intervention score) and subsequently compared between participants of the two intervention groups. A negative change score indicates a change to a utilitarian reasoning and utilitarian decision-making, while a positive change score represents a change to a non-utilitarian reasoning and non-utilitarian decision-making.

### Statistics

All analyses were performed with SPSS 22 (SPSS Inc., Chicago, IL, USA). To analyse whether participants of the two intervention groups differed in physiological and psychological measures at baseline and after the cycling intervention, unpaired *t*-tests were performed. To analyse the effects of moderate and high intensity on moral reasoning (rating change score) and moral decision-making (decision change score, decision time change score) during exercise, mixed-design analyses of covariance with the between-subjects factor exercise intensity (moderate intensity vs. high intensity), and the within-subjects factor dilemma type (personal vs. impersonal) were performed. Cortisol levels were considered as covariates in these analyses to control for differences in individual cortisol response or possible threshold effects of physical exercise (Hill et al., 2008; Gatti and De Palo, 2011). Cigarette consumption per week was considered as additional covariate to control for differences in smoking-related exhaustion effects of physical exercise and dopamine release (Brody et al., 2004; Crockett et al., 2015; North et al., 2015). Of note, some variables were log-transformed (decision times) or root-squared (cortisol levels) before the analyses to account for deviations from normal distribution (Kobayashi and Miyazaki, 2015). The significance level for all analyses was set at *p* ≤ 0.05 and, if necessary, corrected for multiple comparisons using the Bonferroni-method. Given the novelty of our study and its explorative character, we not only considered significant but also marginally significant effects in our analyses. Significance values with a *p* ≤ 0.05 indicate significant effects and significance values with a *p* ≤ 0.10 indicate significant trends for effects. To facilitate the interpretation of these effects, we determined effect size estimates (η_*p*_^2^, *d*) in addition to the significance value (*p*; Cohen, 1988).

## Results

### Participants' characteristics

With the exception of a small difference in body weight, there were no differences in body height, age, moral orientation, or psychopathology between participants of the two intervention groups (see Table 2). On day one, participants of the two intervention groups did also not differ in resting HR and peak HR during the incremental exercise test (see Table 2). At least two of the widely accepted attainment criteria for maximal VO_2_, e.g., an respiratory exchange ratio >1.15, HR ± 10 bpm of age predicted maximum and blood lactate concentration >8 mmol/L, were met by all participants at the end of the incremental test on day one, speaking for a high motivation and valid assessment of participants' physical fitness (Howley et al., 1995; Beltz et al., 2016). Based on their relative VO_2_peak values, all participants could be classified as physically fit (De Pauw et al., 2013).

**Table 2:** Mean (M) and standard deviation (SD) of participants' characteristics assessed at the preparation day for the moderate and high intensity group, respectively.

	**Moderate intensity group (*n* = 16) *M* (*SD*)**	**High intensity group (*n* = 16) *M* (*SD*)**	**Test statistic**
Age (years)	25.9 (3.8)	26.3 (3.6)	*t*_(30)_ = 0.23, *p* = 0.820
Weight (kg)	76.0 (7.8)	82.9 (10.2)	*t*_(30)_ = 2.09, *p* = 0.045
Height (m)	1.81 (0.05)	1.82 (0.06)	*t*_(30)_ = 0.47, *p* = 0.643
VO_2peak_ (mL·min^−1^·kg^−1^)	57.7 (8.8)	56.0 (6.1)	*t*_(30)_ = 0.61, *p* = 0.547
Resting HR (bpm)	73.6 (9.5)	74.6 (12.9)	*t*_(30)_ = 0.24, *p* = 0.811
Peak HR (bpm)	184.3 (9.1)	183.7 (10.4)	*t*_(30)_ = 0.17, *p* = 0.862
EPQ-20 idealism	6.3 (0.7)	5.9 (1.2)	*t*_(30)_ = 1.28, *p* = 0.211
EPQ-20 relativism	6.2 (1.1)	5.9 (0.9)	*t*_(30)_ = 0.98, *p* = 0.337
BSI-18 GSI	0.3 (0.3)	0.3 (0.2)	*t*_(30)_ = 0.59, *p* = 0.560

*VO_2peak_, peak oxygen uptake; HR, heart rate; EPQ, Ethical Positions Questionnaire (Forsyth, 1980); BSI-18, Brief Symptom Inventory; 18, Global Severity Index (Franke et al., 2017)*.

**Figure 2:**
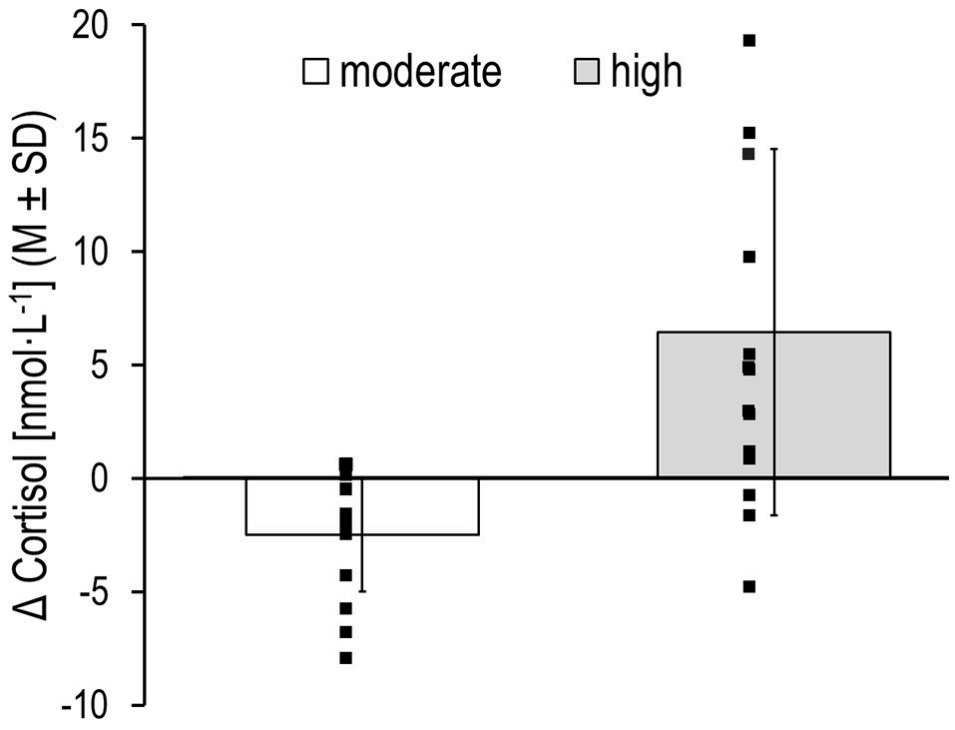
Changes in salivary cortisol levels (Δ = intervention – baseline change) for participants after moderate and high intensity cycling, individual values, and means (M) ± standard deviations (SD).

On day two, there were also no differences in HR, %HR, and cortisol between participants of the two intervention groups for baseline cycling (see Table 3). However, after cycling at different exercise intensities, participants of the two intervention groups showed marked differences in %HR-, blood lactate-, and RPE-values that corresponded to reference ranges of either moderate or high intensity exercise and strain (Garber et al., 2011). There were also marked differences in cortisol levels between participants of the intervention groups as well as a high variability of cortisol levels within participants of the high intensity group (see Table 3 and Figure 2), indicating individual cortisol responses and exercise threshold effects (Hill et al., 2008; Gatti and De Palo, 2011). Furthermore, there were pronounced differences in participants' self-reports of effort, pain, fatigue, mood, wakefulness and arousal (Table 4), indicating that our intervention successfully led to exercise-related effects on the physiological and psychological level (manipulation check).

**Table 3:** Mean (M) and standard deviation (SD) for participants' physiological responses to baseline (cycling at 10% VO_2_peak power output), moderate (cycling at 50% VO_2_peak power output), and high intensity (cycling at 90% VO_2_peak power output) exercise.

	**Moderate intensity group (*n* = 16) *M* (*SD*)**	**High intensity group (*n* = 16) *M* (*SD*)**	**Test statistic**
Baseline HR (bpm)	92.6 (11.3)	95.9 (11.7)	*t*_(30)_ = 0.80, *p* = 0.430
Baseline %HR (% of HR_peak_)	50.3 (6.0)	52.1 (5.1)	*t*_(30)_ = 0.93, *p* = 0.361
Baseline salivary cortisol (nmol·L^−1^)	9.5 (3.7)	9.1 (4.1)	*t*_(30)_ = 0.41, *p* = 0.686
Intervention HR (bpm)	130.8 (17.2)	175.9 (11.9)	*t*_(30)_ = 8.34, *p* < 0.001
Intervention %HR (% of HR_peak_)	70.8 (6.9)	95.8 (4.3)	*t*_(30)_ = 11.89, *p* < 0.001
Intervention salivary cortisol (nmol·L^−1^)	7.0 (2.3)	15.4 (8.8)	*t*_(30)_ = 3.93, *p* < 0.001
Intervention blood lactate concentration (mmol·L^−1^)	2.4 (1.2)	9.5 (3.0)	*t*_(30)_ = 8.79, *p* < 0.001

*HR, heart rate; %HR, percentage of individual peak HR*.

**Table 4:** Mean (M) and standard deviation (SD) for participants' psychological responses to cycling at moderate or high intensity that were immediately assessed after exercise cessation.

	**Moderate intensity group (*n* = 16) *M* (*SD*)**	**High intensity group (*n* = 16) *M* (*SD*)**	**Test statistic**
BORG-RPE effort	12.1 (2.0)	18.9 (0.9)	*t*_(30)_ = 12.24, *p* < 0.001
POMS-F fatigue	9.2 (6.8)	25.3 (7.3)	*t*_(30)_ = 6.45, *p* < 0.001
CR-10 pain	1.6 (1.0)	7.3 (2.1)	*t*_(30)_ = 9.61, *p* < 0.001
MDMQ mood	17.4 (1.4)	15.1 (2.8)	*t*_(30)_ = 2.96, *p =* 0.003
MDMQ wakefulness	16.4 (2.2)	12.2 (2.6)	*t*_(30)_ = 4.99, *p* < 0.001
MDMQ arousal	17.4 (1.5)	13.9 (2.9)	*t*_(30)_ = 4.27, *p* < 0.001

*BORG-RPE, Borg Scale-Rating of Perceived Effort (Borg, 1990; Marcora, 2010; Pageaux, 2016); POMS-F, Profile of Mood State-Fatigue (Albani et al., 2005); CR-10, Perceived Pain (Cook et al., 1997); MDMQ, Multidimensional Mood Questionnaire (Hinz et al., 2012)*.

### Moral reasoning

**Figure 3:**
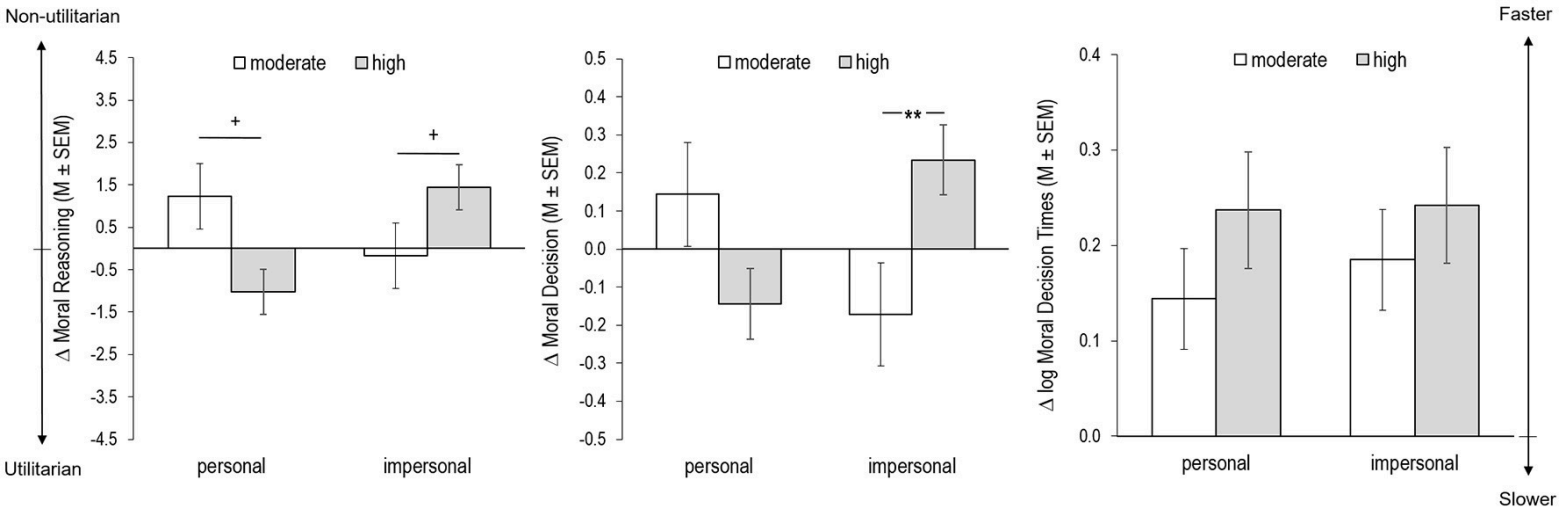
Change scores (Δ = baseline – exercise) for moral reasoning, moral decision-making, and moral decision-making time differentiated for personal and impersonal dilemmas for participants during moderate and high intensity cycling. Bars represent Mean (M) and Standard Error of the Mean (SEM); ^+^*p* ≤ 0.10, ^*^p ≤ 0.05, ^**^p ≤ 0.01 (Bonferroni-corrected *post hoc* tests).

No significant effect on the change in moral permissibility was found for dilemma type [*F*_(28, 1)_ = 0.308, *p* = 0.584, η_*p*_^2^ = 0.011] and exercise intensity [*F*_(28, 1)_ = 0.142, *p* = 0.710, η_*p*_^2^ = 0.005]. However, the interaction of dilemma type and exercise intensity had a significant effect on the moral reasoning change scores [*F*_(28, 1)_ = 9.397, *p* = 0.005, η_*p*_^2^ = 0.251, see Figure 3]. Bonferroni-corrected *post hoc* tests revealed a marginally significant antagonistic change of moral reasoning in participants of the two intervention groups on the personal [*p* = 0.079] and the impersonal [*p* = 0.069] dilemmas. For the personal dilemmas, participants of the moderate intensity group tended to non-utilitarian reasoning (positive change), while participants of the high intensity group tended to utilitarian reasoning (negative change). For the impersonal dilemmas, participants of the high intensity group tended to non-utilitarian reasoning (positive change), whereas participants of the moderate intensity group neither tended to utilitarian nor non-utilitarian reasoning.

### Moral decision-making

The change in moral decision-making was neither significantly affected by dilemma type [*F*_(28, 1)_ = 0.154, *p* = 0.697, η_*p*_^2^ = 0.005] nor by exercise intensity [*F*_(28, 1)_ = 0.239, *p* = 0.629, η_*p*_^2^ = 0.008] but again by an interaction of dilemma type and exercise intensity [*F*_(28, 1)_ = 5.911, *p* = 0.022, η_*p*_^2^ = 0.174, see Figure 3]. Bonferroni-corrected *post hoc* test revealed differences in moral decision-making on impersonal [*p* = 0.010] but not on personal [*p* = 0.199] dilemmas between participants of the two intervention groups. For the impersonal dilemma, participants in the high intensity group showed a strong tendency for non-utilitarian moral decision-making (large positive change), whereas participants in the moderate intensity group tended to utilitarian decision-making (negative change).

### Moral decision-making time

No significant effect on the change in decision-making time was found for dilemma type [*F*_(28, 1)_ = 0.428, *p* = 0.518, η_*p*_^2^ = 0.015], exercise intensity [*F*_(28, 1)_ = 0.745, *p* = 0.395, η_*p*_^2^ = 0.026] or their interaction [*F*_(28, 1)_ = 0.405, *p* = 0.530, η_*p*_^2^ = 0.014; see Figure 3].

## Discussion

In the present study, we were able to demonstrate that different individually-tailored exercise intensities led to different levels of physical exertion and fatigue that had divergent effects on moral-reasoning and moral decision-making on personal and impersonal dilemmas. High intensity exercise facilitated non-utilitarian reasoning and non-utilitarian decision making on impersonal dilemma. Moderate intensity exercise, on the contrary, did not affect reasoning on impersonal dilemmas in a utilitarian or non-utilitarian way but facilitated utilitarian decision making on impersonal dilemmas. It should be noted, however, that the effects of exercise on moral decision-making were more pronounced than the effects of exercise on moral reasoning. As some of these effects were only marginally significant after applying the Bonferroni-correction for multiple comparisons, these effects should be treated with caution. It should be kept in mind, however, that our study is the first one to investigate these types of effects, which may justify a liberal rather than conservative approach to data analysis and data interpretation. Notwithstanding the exploratory nature of our study, we clearly recommend a further investigation of these effects in future studies.

Dual process theories in conjunction with fatigue-based neuro-cognitive theories provide plausible explanations for the exercise-induced changes in moral reasoning and moral decision-making that we found in the present study (Baumeister et al., 1998; Schmeichel et al., 2003; Vohs et al., 2008; Pattyn et al., 2018; Schmit and Brisswalter, 2018). The parallel execution of a motor and a moral dilemma task during high physical exertion may have caused fatigue- or exertion-induced limitations in prefrontally mediated executive resources and executive functioning, thereby favoring fast and intuitive thinking rather than slow and deliberative thinking. Considering that non-utilitarian reasoning and decision-making is more in line with common and internalized social norms, it depends less on executive resources and executive functioning than utilitarian-reasoning and decision-making. As a consequence, non-utilitarian reasoning and decision-making are more likely to emerge under fast and intuitive thinking than utilitarian reasoning and decision-making. This may explain why participants were more likely to engage in non-utilitarian reasoning and non-utilitarian decision-making after high intensity exercise that induced more physical effort and fatigue than moderate intensity exercise. Of note, similar changes in non-utilitarian responding have been found after cognitive fatigue in a previous study (Timmons and Byrne, 2018). Interestingly, these changes have only been found on impersonal but not personal dilemmas, which was also the case in the present study. We can only speculate why exertion or fatigue changed participants' performance on the impersonal as compared to the personal dilemmas in the present and previous study. It may be possible that personal dilemmas forced a distinct thinking style that was not further affected by exertion or fatigue in both studies. It may also be possible that exertion or fatigue induced a self-serving bias that prevents ego-threating regrets about taking the life of one individual for the lives of five individuals, thereby facilitating non-utilitarian reasoning and non-utilitarian decision-making under fatigue on personal rather than impersonal dilemmas. Clearly, more research is needed to understand why fatigue and exertion only affected decision-making on impersonal but not personal dilemmas in both studies. Taking into account that these studies differed markedly in study design (induction of cognitive rather than physical fatigue, task performance after rather than during fatigue induction etc.), the similarities of findings are striking, indicating that dual process theories provide a similar plausible explanation for changes on moral dilemma tasks as on other tasks that rely on executive resources and executive functioning (Baumeister et al., 1998; Capraro and Cococcioni, 2016; Rand, 2016). Moreover, these studies suggest that various manifestations of fatigue, induced by cognitive and/or physical interventions, may lead to limitations of executive resources, executive functioning and self-control that either alone or in concert account for changes in moral reasoning and moral decision-making. In this respect, it is important to note that it was probably not the physical intervention *per se* but the intervention-induced exertion with its associated symptoms of fatigue and effort that was responsible for the observed changes in moral reasoning and moral decision-making in the present study (Vohs et al., 2008; Pattyn et al., 2018; Schmit and Brisswalter, 2018).

The aforementioned explanations for exercise-induced changes in moral reasoning and moral decision-making in the context of dual process theories complement those explanations that can be derived from psychobiological theories on exercise-induced hypofrontality (Dietrich, 2006; Dietrich and Audiffren, 2011). According to these theories, it may be possible that the moral reasoning and moral decision-making during high intensity cycling led to competing activity changes between regions that are relevant for the execution of motor and cognitive tasks (Doya, 2000; Poldrack and Packard, 2003; Dietrich, 2006; Dietrich and Audiffren, 2011; Pascual et al., 2013). These competing activity changes may have caused hypo-activation in prefrontal brain regions that are relevant for the allocation of executive resources and executive functioning. These prefrontally mediated limitations in executive resources and executive functioning may have impaired deliberative thinking and facilitate intuitive thinking, thereby favoring non-utilitarian rather than utilitarian reasoning and decision-making. Moreover, deliberative thinking that is relevant for utilitarian reasoning and utilitarian decision-making may have been further impaired by physical effort and/or demotivation. During high-intensity exercise the severity of sensations of muscle pain, dyspnoea, other somatic markers or effort perception may have triggered a switch from an sub-aware completion of the motor task (like during moderate-intensity cycling) to a conscious attempt to regulate and maintain motor behavior (Edwards and Polman, 2013). Hypothesizing that effort, no matter if physical or mental, is a limited resource, active engaging in a physically demanding task and regulating the respective motor activity may have depleted the same prefrontally mediated executive resources that were used for deliberative reasoning and decision-making. In this respect, motivation may play a role in maintaining attention for cognitive tasks by up-regulation of arousal (Schmit and Brisswalter, 2018). It may be argued that participants that were physically exhausted or invested high effort in the concurrent physical task were less motivated or invested less effort to try to think about the moral dilemmas (Baumeister et al., 1998; Vohs et al., 2008). However, it has recently been shown that mentally fatigued participants tended to judge reasoning about moral dilemmas more difficult than non-fatigued participants, suggesting that the participants at least attempted to think about the dilemmas (Timmons and Byrne, 2018). Our findings of similar changes in decision times for participants of the high and moderate intensity group do support these observations.

Although we strongly believe that dual process theories and exercise-dependent hypofrontality theories provide the most plausible explanation for the fatigue-related changes in moral reasoning and moral decision-making in the present study, we nonetheless discuss other explanations in the following. Considering that moral reasoning and moral decision-making is susceptible to mood changes, it may be possible that mood changes also affected participants' performance on the moral dilemma task. For instance, a positive mood induction has been shown to increase the likelihood of utilitarian responding on personal dilemmas (Valdesolo and DeSteno, 2006). In this regard, it is noteworthy that participants in the high intensity group reported a slightly more negative mood than participants in the moderate intensity group as indicated by the respective difference in MDMQ values. It may, thus, be possible that participants' negative mood in the high intensity group accounted for the decrease in utilitarian reasoning and utilitarian decision-making on impersonal dilemmas and that participants' positive mood in the moderate intensity group accounted for an increase in utilitarian reasoning and utilitarian decision-making on impersonal dilemmas. Besides mood, psychopathology may have also affected participants' performance on the moral dilemma task. For instance, anxiety has been shown to decrease the likelihood of utilitarian responding on impersonal dilemmas (Whitton et al., 2014). However, participants in the two intervention groups did not differ in psychopathology as indicated by the BSI values, implying that psychopathology did not affect participants' moral reasoning and moral decision-making. Of note, personality traits associated with participants' moral orientation did also not differ between participants of the two intervention groups as indicated by the EPQ values, ruling out that differences in moral orientation affected participants' moral reasoning and moral decision-making.

Before drawing final conclusions, the following methodological aspects should be considered. A strength of our study is the approach of using acute endurance exercise as a model to investigate immediate, contemporaneous effects of exertion and fatigue on moral reasoning and moral decision-making. Participants in our study performed the moral dilemma task *during* different levels of physical exertion. Further, we compared *individual* transgressions in response to an *individualized* physical workload leading to different levels of exertion and fatigue. However, our study also has some weaknesses. It was designed on basis of the model of hypofrontality (Dietrich, 2006; Dietrich and Audiffren, 2011) that links profound changes in prefrontal brain regions with exercise intensity. However, recently it has been proposed that not exercise intensity *per se* but the level of exertion and fatigue may lead to cognitive changes (Schmit and Brisswalter, 2018). Despite using individualized exercise intensity based on individual peak performance to induce homogeneous levels of effort perception and perceived fatigability in all participants, individual changes in the activation of brain regions involved in solving moral dilemmas may have confounded the findings by increasing the variance of the moral change scores. Further, our hypothesis was based on the assumption that a decrease in the permissibility to sacrifice the life of an individual for the life of five other individuals reflects non-utilitarian, that is, deontological reasoning and decision-making across all participants. A decrease in permissibility is, thus, assumed to reflect a change to a more intuitive reasoning and decision-making. This is because solving a moral dilemma from a deontological position, bases on (internalized) ethical rules, where action is more important than its consequences. However, while the common distinction between utilitarian and deontological considerations and decision-making options initially provides methodologically verifiable scenarios, it has to be critically examined with regard to future work. Prima facie, both terms are only references to the way ethical decisions are made, not to what content (e.g., value) is preferred. By no means does a utilitarian decision necessarily has to be altruistically motivated (even if it is most of the time). The same applies vice versa to a deontological perspective that can be selfish, but by no means necessarily so, because it only means that it depends solely on intrinsic reasons, but it does not say that these grounds for decision have to pursue subjective values only (Kant, 2003). It should also be borne in mind that the common alternative of deontological and utilitarian ethics used in this study and elsewhere is not comprehensive. In philosophy, on the contrary, teleological ethics are differentiated from deontological ethics, and very often these two are distinguished yet again from virtue ethics (MacIntyre, 2007). Utilitarianism is only a particular form of teleological, which is, purpose-oriented ethics. Therefore, if moral reasoning or moral decision-making is to be explored, it would be necessary to differentiate more strongly the categories of analysis. In particular, it would be worth considering to what extent virtue ethics are principally relevant to the analysis of stress situations, inasmuch as such an ethic involves the idea that moral reasoning depends on habitual characteristics, only secondarily on the consideration and decision-making processes (Brodie, 1991). It is also of note that the assumptions that can be made on basis of our findings can only be applied to male participants because we did not investigate female participants. Previous findings imply that female participants show more deontological reasoning on moral dilemmas than male participants and that cognitive fatigue interacts with participants' gender in the context of altruism (Fumagalli et al., 2010; Friesdorf et al., 2015; Rand et al., 2016; Capraro and Sippel, 2017). It, thus, seems worthwhile to not only investigate how the interaction of physical exhaustion and fatigue with participants' gender affects moral reasoning and moral decision-making but also to investigate how other features of moral dilemmas, beyond the personal-impersonal distinction, affect moral reasoning and moral decision-making.

To sum up, we first introduced a real-world example of moral reasoning and moral decision-making under physical exertion and fatigue to highlight that our study is of practical relevance. The real-world example suggests that physical exertion and fatigue may affect reasoning and decision-making on moral dilemmas that are related to violations of the moral principle to cause no harm. Our empirical findings on hypothetical moral dilemmas partially support this suggestion by showing that high intensity exercise rather than moderate intensity exercise tends to facilitate non-utilitarian reasoning and non-utilitarian decision-making on impersonal dilemmas. Although these findings are in accordance with fatigue-based neurocognitive process theories (Baumeister et al., 1998; Vohs et al., 2008; Capraro and Cococcioni, 2016; Rand, 2016; Schmit and Brisswalter, 2018), they should be considered as preliminary until replicated and extended in future studies that use more ecologic valid moral dilemma tasks to investigate moral reasoning and moral-decision making in male as well as female participants.

## Author contributions

MW and AL designed this study. MW and MR collected the data. MW and AL analyzed and interpreted the data. MW and AL drafted the manuscript. SK contributed to the writing. All authors reviewed and approved the final version.

### Conflict of interest statement

The authors declare that the research was conducted in the absence of any commercial or financial relationships that could be construed as a potential conflict of interest.

## Footnotes

^1^https://www.youtube.com/watch?v=c1w0g6eNxMk
