# Supplementary material for: It's Harder to Push, When I Have to Push Hard—Physical Exertion and Fatigue Changes Reasoning and Decision-Making on Hypothetical Moral Dilemmas in Males
Source: Front Behav Neurosci. 2018 Nov 23;12:268. doi: 10.3389/fnbeh.2018.00268 (PMC6276357; doi:10.3389/fnbeh.2018.00268)
Supplement: Supplementary file 1 [file Data_Sheet_1.docx]

**Supplement_ Weippert et al. 2018 Front Behav Neurosci**

**Translated example for personal dilemma: “EXIT”**

*You're in a stadium as terrorists detonate a bomb and shoot wildly around you. Together with five other spectators you flee to one of the emergency exits. However, you won't get far because the way to the emergency exit is blocked by a pile of rubble. A spectator already tries to crawl through a hole in the pile of rubble to get to the emergency exit. The terrorists will catch up with you any moment and shoot at you. You and the other spectators will die if you don't get into the emergency exit immediately. If you jump around on the pile of rubble, an avalanche of rubble will come loose and the way to the emergency exit will be uncovered. The spectator will be buried and die. But you will save yourself and the other five spectators from the terrorists.*

*To what extent is it morally reprehensible or morally permissible for you to jump around on the pile of rubble to save you and the five other spectators from the terrorists?*

*Would you jump around the pile of rubble to save yourself and the five other spectators from the terrorists?*

**Translated Example for an impersonal dilemma: “LIFT”**

*You got stuck with five other spectators in an elevator in the stadium. A spectator just climbs out of the door to get to safety via a ladder in the shaft. There you see that the ropes on which the elevator is hanging are damaged and are about to break. You and the other five spectators will die unless the elevator is immediately prevented from crashing. If you push the door button, the door will close quickly and the spectator will fall into the shaft. The elevator will become lighter and the ropes on which the elevator hangs will not break. The spectator will suffer serious injuries and die as a result of the fall. But you will save yourself and the other five spectators from the lift crash.*

*To what extent is it morally reprehensible or morally permissible for you to push the door button to save yourself and the five other spectators from the crash?*

*Would you push the door button to save yourself and the other five spectators from the crash?*
